# Characterization of the expression of gastrin-releasing peptide and its receptor in the trigeminal and spinal somatosensory systems of Japanese macaque monkeys: Insight into humans

**DOI:** 10.1002/cne.25376

**Published:** 2022-06-10

**Authors:** Keiko Takanami, Takumi Oti, Yasuhisa Kobayashi, Koki Hasegawa, Takashi Ito, Naoaki Tsutsui, Yasumasa Ueda, Earl Carstens, Tatsuya Sakamoto, Hirotaka Sakamoto

**Affiliations:** 1 Ushimado Marine Institute (UMI), Okayama University, Okayama, Japan; 2 Department of Genetics, Mouse Genomics Resources Laboratory, National Institute of Genetics, Sokendai (The Graduate University for Advanced Studies), Shizuoka, Japan; 3 Department of Anatomy and Neurobiology, Kyoto Prefectural University of Medicine, Kyoto, Japan; 4 Department of Neurobiology, Physiology, and Behavior, University of California, Davis, California, USA; 5 Department of Biological Sciences, Faculty of Science, Kanagawa University, Kanagawa, Japan; 6 Department of Aquatic Biology, Fisheries, Faculty of Agriculture, Kindai University, Nara, Japan; 7 Center for Instrumental Analysis, Kyoto Pharmaceutical University, Kyoto, Japan; 8 Theranostic Pharmaceuticals Laboratory, Department of Radiological Sciences, School of Health Sciences, Fukushima Medical University, Fukushima, Japan; 9 Department of Marine Bioresources, Mie University, Mie, Japan; 10 Department of Physiology, Kyoto Prefectural University of Medicine, Kyoto, Japan; 11 Department of Physiology, Kansai Medical University, Osaka, Japan

**Keywords:** gastrin-releasing peptide, gastrin-releasing peptide receptor, itch, ligand derivative stain, macaque monkey, primates, trigeminal sensory system

## Abstract

Gastrin-releasing peptide (GRP) and its receptor (GRPR) have been identified as itch mediators in the spinal and trigeminal somatosensory systems in rodents. In primates, there are few reports of GRP/GRPR expression or function in the spinal sensory system and virtually nothing is known in the trigeminal system. The aim of the present study was to characterize GRP and GRPR in the trigeminal and spinal somatosensory system of Japanese macaque monkeys (*Macaca fuscata*). cDNA encoding *GRP* was isolated from the macaque dorsal root ganglion (DRG) and exhibited an amino acid sequence that was highly conserved among mammals and especially in primates. Immunohistochemical analysis demonstrated that GRP was expressed mainly in the small-sized trigeminal ganglion and DRG in adult macaque monkeys. Densely stained GRP-immunoreactive (ir) fibers were observed in superficial layers of the spinal trigeminal nucleus caudalis (Sp5C) and the spinal cord. In contrast, GRP-ir fibers were rarely observed in the principal sensory trigeminal nucleus and oral and interpolar divisions of the spinal trigeminal nucleus. cDNA cloning, in situ hybridization, and Western blot revealed substantial expression of *GRPR* mRNA and GRPR protein in the macaque spinal dorsal horn and Sp5C. Our Western ligand blot and ligand derivative stain for GRPR revealed that GRP directly bound in the macaque Sp5C and spinal dorsal horn as reported in rodents. Finally, GRP-ir fibers were also detected in the human spinal dorsal horn. The spinal and trigeminal itch neural circuits labeled with GRP and GRPR appear to function also in primates.

## 1 | INTRODUCTION

Gastrin-releasing peptide (GRP), a 27 amino acid peptide isolated from the porcine intestine, is distributed throughout the central nervous system (CNS) and gastrointestinal tract of mammals (Panula et al., 1988). GRP selectively binds with high affinity to the GRP receptor (GRPR), a member of the bombesin receptor family (Jensen et al., 2008). GRP and GRPR play roles in many physiological functions of the CNS including food intake (Ladenheim et al., 1996), circadian rhythms (Shinohara et al., 1993), anxiety or fear responses (Merali et al., 2006), and male sexual function (Sakamoto et al., 2008).

Furthermore, the GRP-GRPR system has been discovered to mediate itch in the mouse spinal somatosensory system (Chen, 2021; Sun & Chen, 2007; Sun et al., 2009). Intrathecal injection of a GRPR antagonist reduced the scratching behavior and selective ablation of GRPR-expressing neurons in the spinal dorsal horn abolished scratching behaviors in mice (Sun et al., 2009). Histological analyses in rodents revealed that GRP-immunoreactivity is localized in the small- and medium-sized neurons in the spinal dorsal root ganglion (DRG), trigeminal ganglion (TG), and spinal dorsal horn (Albisetti et al., 2019; Barry et al., 2020; Bell et al., 2020; Chen, 2021; Fleming et al., 2012; Sun & Chen, 2007; Sun et al., 2009; Takanami & Sakamoto, 2014; Takanami et al., 2016; Takanami et al., 2014). GRP-containing fibers are observed in the superficial layers of the spinal dorsal horn and caudal part of the trigeminal nucleus (Sp5C) of rats and the Asian house musk shrew (suncus) as well as mice (Albisetti et al., 2019; Barry et al., 2020; Bell et al., 2020; Sun & Chen, 2007; Sun et al., 2009; Takanami et al., 2016; Takanami et al., 2014). Also, GRPR is localized in the spinal dorsal horn and Sp5C in rodents (Kiguchi et al., 2020; Sun & Chen, 2007; Sun et al., 2009; Takanami et al., 2016).

Our previous study demonstrated in macaques that GRP-positive fibers in the sacral autonomic nucleus, which controls penile function, showed male-specific dimorphism, but that GRP-positive fibers in the dorsal horn of the lumbosacral spinal cord controlling itch transmission appeared to show no sexual dimorphism (Ito et al., 2018). Macaques suffering from idiopathic chronic itch exhibited a relatively higher severity of scratching and also consistently displayed an increase in the percentage of GRP-immunopositive nerve fibers at the dermal-epidermal junction of lichenified skin, correlated with the scratching behavior (Nattkemper et al., 2013). Thus, it has been suggested that GRP is involved in the transmission of itch in the spinal somatosensory system of primates. However, little information exists on the GRP-GRPR pathway in the trigeminal somatosensory system, which transmits the information of orofacial sensation in primates, although it is clinically important to know whether the GRP-GRPR pathway exists and has similar functions in the trigeminal system. Macaques are considered as excellent model because they are closely related to humans. Therefore, in this study, we sought to identify GRP and GRPR in the trigeminal somatosensory system of primates using Japanese macaque monkeys *(Macaca fuscata*) and human tissues. In addition, we analyzed whether GRP binds to GRPR in the itch circuit of macaques by Western ligand blot and ligand derivative staining methods.

## 2 | MATERIALS AND METHODS

### 2.1 | Animals

A total of 11 male and female Japanese macaque monkeys, *M. fuscata* (n = 5 males: aged 2-, 3-, 7-, 9, and 10-year-old, weight 2.3–12.6 kg; and n = 6 females: aged 8-, 10-, 10-, 10-, 10-, and 11-year-old, 7.2–10.8 kg), were used in this study. Macaque monkeys were maintained in a temperature-controlled (22–24°C) room under a daily photoperiod of 12/12 h light/dark cycle (lights on 0800 h-2000 h). We confirmed these animals were free of specific pathogens. Food and water was available ad libitum. All animals were kept in individual cages. The experimental protocols followed the guidelines of the Ministry of Education, Culture, Sports, Science and Technology (MEXT) of Japan and were approved in accordance with the Guide for the Care and Use of Laboratory Animals prepared by Okayama University (Okayama, Japan), by the National Institute of Genetics (Shizuoka, Japan), by Kyoto Prefectural University of Medicine (Kyoto, Japan), by Kyoto Pharmaceutical University (Kyoto, Japan), and by Kansai Medical University (Osaka, Japan). All efforts were made to minimize animal suffering and reduce the number of animals used in this study.

### 2.2 | Partial cloning of macaque monkey *GRP* and *GRPR* genes

Macaques (1 male, 2 females) were sacrificed by exsanguination under deep pentobarbital anesthesia (90 mg/kg i.m.). Cervical DRG and spinal cord were quickly removed and placed on dry ice and were carefully dissected for *GRP* and *GRPR* gene cloning, respectively. Cervical spinal cords were punched out using an 18 G needle. Total RNA of cervical DRG and spinal cord, respectively, was isolated using WaxFree Nucleic Acids Extraction kit (TrimGen, Sparks, MD) and Illustra RNAspin mini RNA isolation kit (GE Healthcare Bio-Science AB, Uppsala, Sweden). The concentration of total RNA samples was measured using Qbit RNA HS kit (Life Technologies, Carlsbad, CA). Two hundred forty nanogram of total RNA was reverse transcribed using random hexamer primers and Omniscript RT kit (Qiagen, Hilden, Germany) according to the manufacturer’s instructions. RT-PCR was performed using primers for GRP (Forward: 5′-gtcctactggcgctggtc-3′, Reverse: 5′-ttccccattaagtgccccac-3′) and for *GRPR* primers (Forward: 5′-tcggctggttgcttctcatc-3′, Reverse: 5′-gcaaccgagtgaagatgaag-3′), and (Forward: 5′-atcatccggtctcacagcac-3′, Reverse: 5′-tacccccacctacaccactc-3′) designed from conserved regions of mammal GRP region. The resulting PCR amplicons for GRP (131 bp) and GRPR (560, 263 bp) were subcloned into pGEM-T easy vector (Promega, Madison, Wl) and sequenced.

### 2.3 | Gene database search and alignment of sequences

**FIGURE 1:**
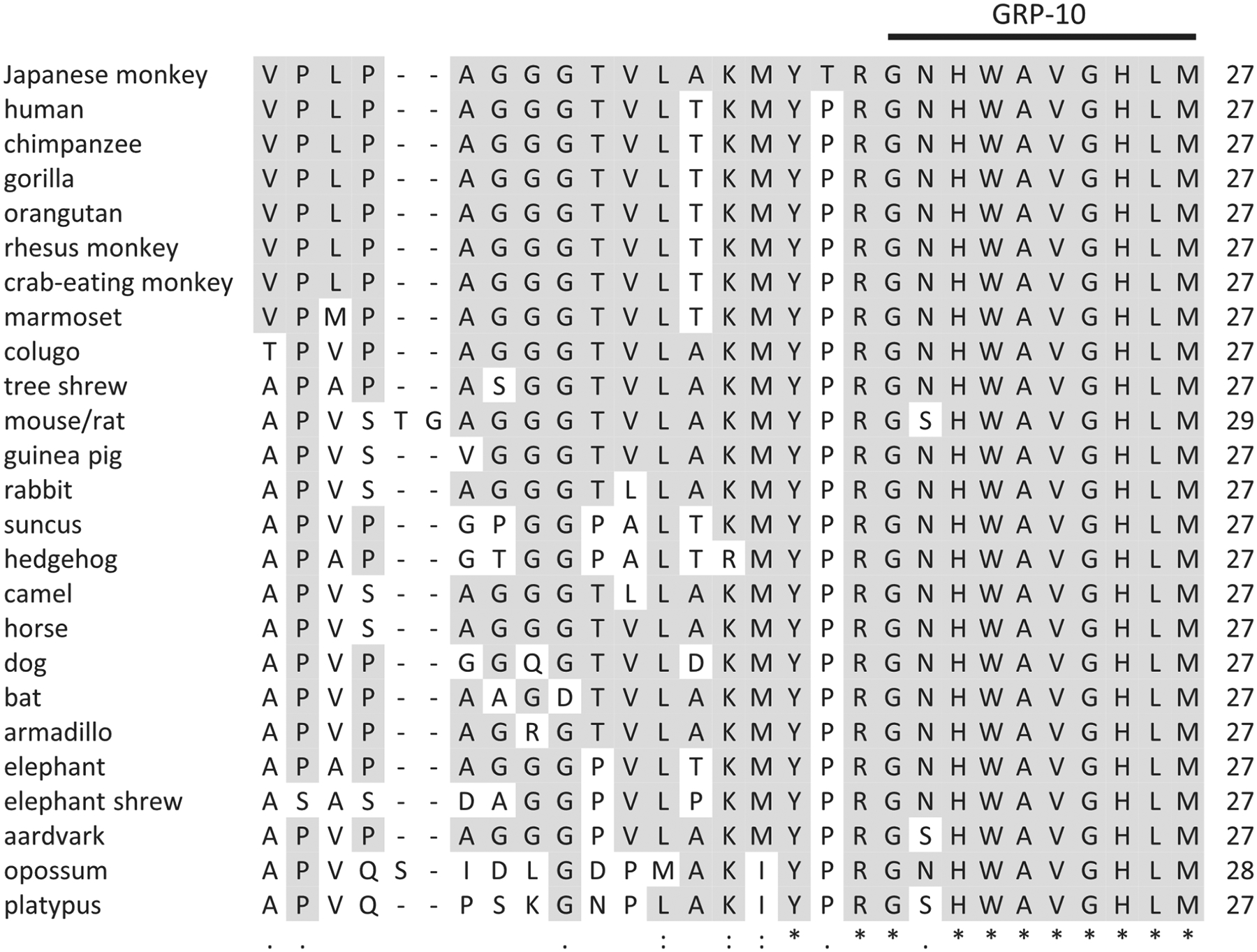
Alignment of the amino acid sequences of GRP of mammals. Gaps were introduced to improve alignment (−). Amino acids identical to those in other species are indicated by asterisks (*). Colon (:) indicates conserved substitution and dot (.) indicates semiconserved substitution. The GenBank accession numbers of GRP are: human (NP_001012531), chimpanzee (XP_001141943), gorilla (XP_004059526.1), orangutan (XP_009250696.1), rhesus monkey (NM_001278448.2), crab-eating monkey (XM_005593048.2), marmoset (XP_002757306.2), colugo (XP.008578400.1), tree shrew (XP.006140000.1), mouse (NM_175012.4), rat (NM_133570.5), guinea pig (XP.003474182), rabbit (XP_008259697.2), suncus (LC138361.1), hedgehog (XP_007529081.1), camel (XP_010978281.1), horse (XP_001489383.1), dog (XP_038509627.1), bat (XP_039696374.1), armadillo (XP_023443420.1), elephant (XP_010584999.1), elephant shrew (XP_006891825.1), aardvark (XP_007933738.1), opossum (XP_007487573), platypus (XP_028915221.1)

Alignment of amino acid sequences of the GRP protein from different species was performed using CLUSTAL format alignment by MAFFT FFT-NS-i (v7.471). Genome and amino acid sequences from different species were obtained from NCBI (www.ncbi.nlm.nih.gov) DNA database. Details of the program settings are given in the legend for Figure 1. CLUSTAL format alignment by MAFFT FFT-NS-i (v7.471) was used to compare the Japanese macaque monkey (*M*. *fuscata) GRPR* we cloned and the *M*. *mulatto GRPR* region (NM_001278448.2) from the NCBI DNA database.

### 2.4 | Tissue preparation for histochemistry

Macaque monkeys (four males, three females) used for immunohistochemistry (IHC) were anesthetized with an overdose of pentobarbital sodium (90 mg/kg i.m.) and transcardially perfused with physiological saline followed by 4% paraformaldehyde in 0.1 M phosphate buffer (PB) (pH 7.4). Brainstem, spinal cords, TGs, and DRGs were quickly removed and immersed in the same fixative overnight at 4°C. After immersion in 25% sucrose in 0.1 M PB at 4°C for cryoprotection until they sank, the preparations were quickly frozen using powdered dry ice and cut into 30 μm cross-sections using a cryostat (CM3050 S, Leica, Nussloch, Germany) and stored in a cryoprotectant (30% glycerol and 30% ethylene glycol in phosphate-buffered saline; PBS) (pH 7.4) at −20°C until use. Macaque monkeys (One male, three females) used for in situ hybridization and ligand binding histochemistry were deeply anesthetized with an overdose of pentobarbital sodium (90 mg/kg i.m.) and transcardially perfused with physiological saline. Spinal cords were quickly removed and then frozen using dry ice and stored at −80°C until use. The preparations were cut into 14 μm cross-sections for in situ hybridization and ligand derivative staining using a cryostat.

### 2.5 | Toluidine blue staining

Thirty micrometer cross-sections of brainstem and spinal cord were mounted in sequence on glass slides. The slides were immersed sequentially for 2 min in 100% ethanol, xylene, 100% ethanol, 90% ethanol, and 70% ethanol. They were then stained with 0.1% toluidine blue in sodium borate buffer for 10 min and dehydrated through a graded ethanol series (70, 80, 90, 100%). The preparations were then soaked in xylene and coverslipped.

### 2.6 | Antibody characterization

**FIGURE 2:**
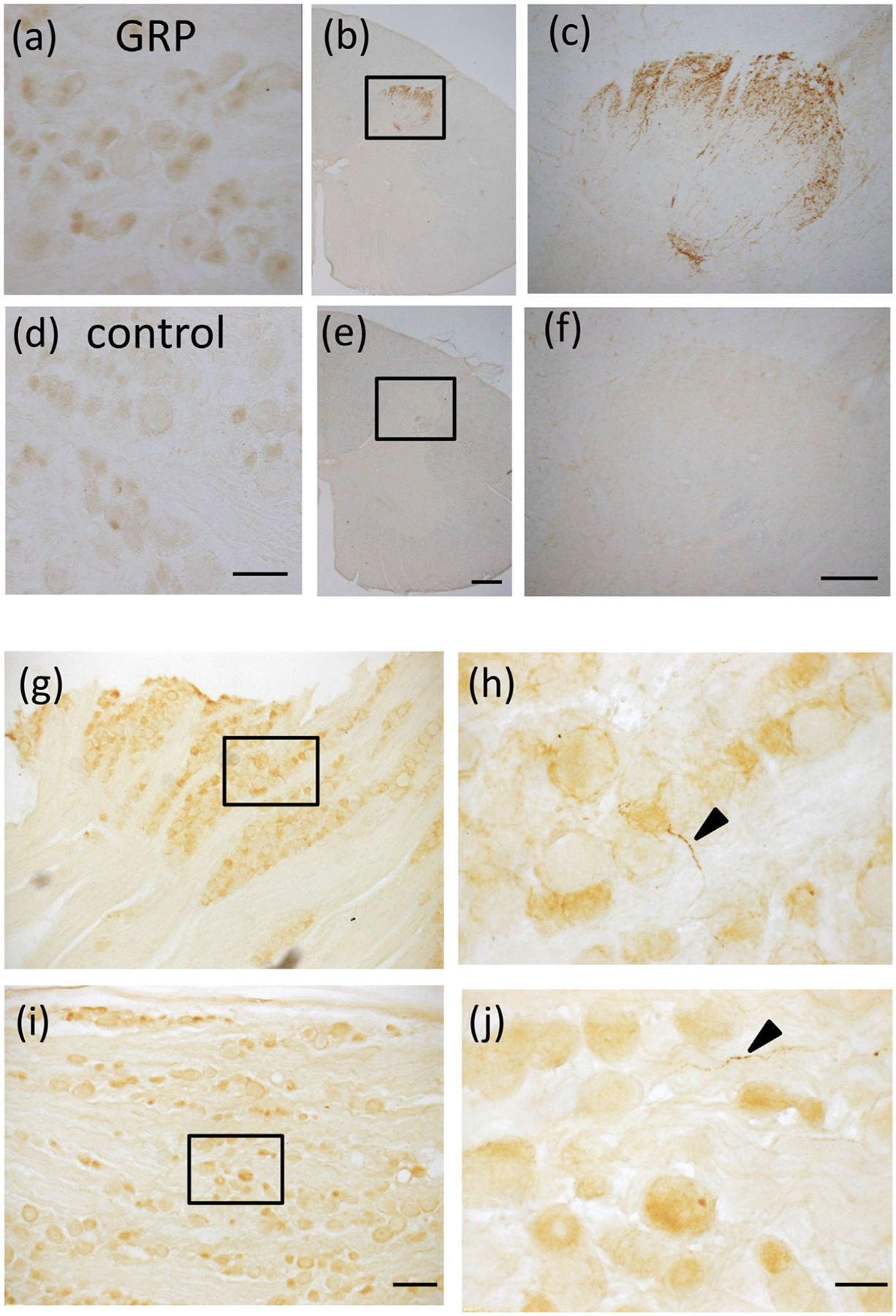

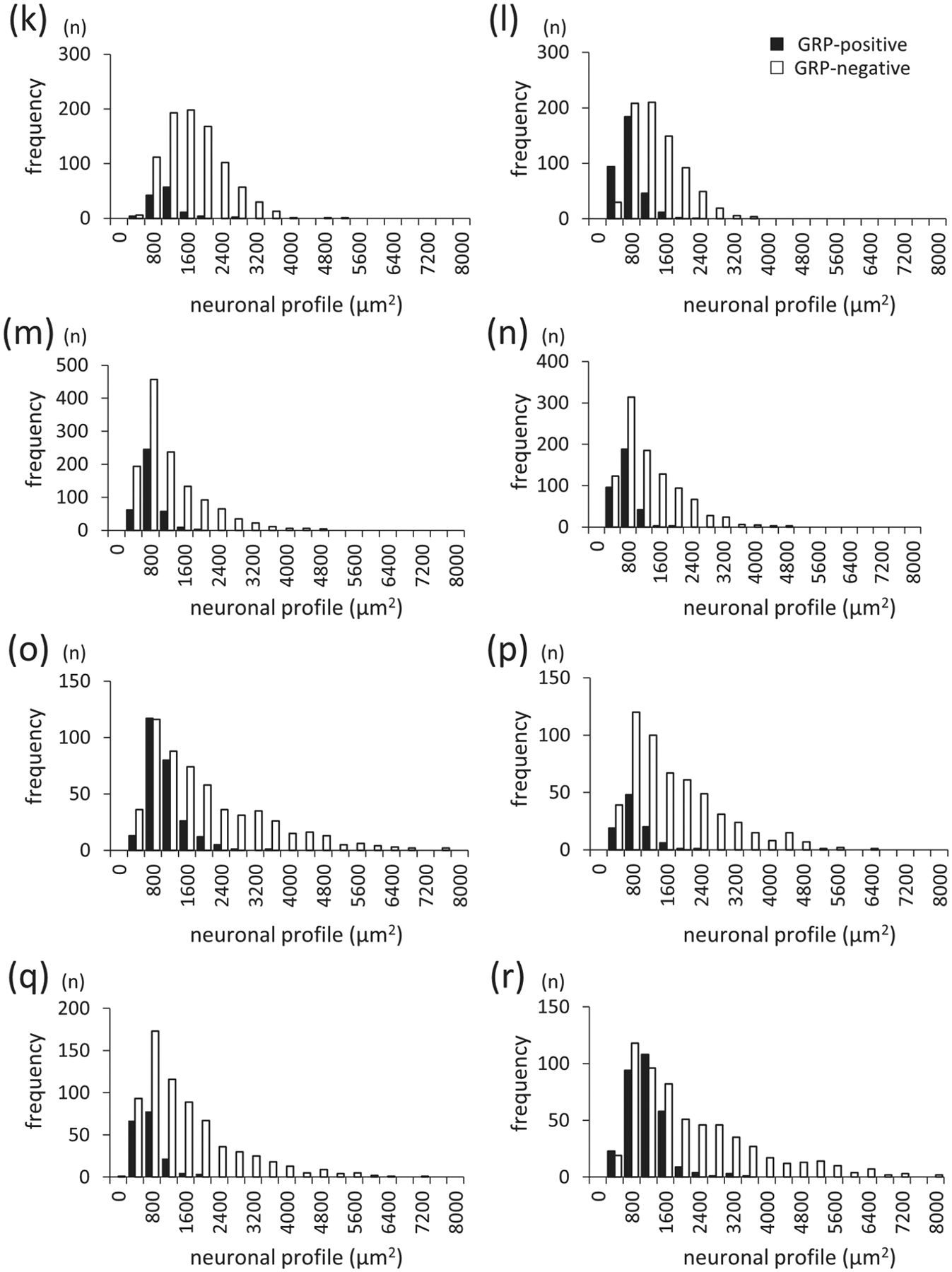
GRP expression and distribution in the trigeminal ganglion (TG) and dorsal root ganglion (DRG) in macaque monkeys, (a–f) Immunohistochemical staining using GRP antiserum in the DRG and cervical spinal cord in adult macaque monkeys. GRP-immunoreactivity (ir) was observed in some neurons in the macaque monkey DRG (a) and in dense fiber projections to the superficial layers of the cervical spinal cord (b, c). Controls in which anti-GRP antiserum was preabsorbed with an excess of antigen peptide (50 μg/mL) showed an absence of GRP expression in the DRG (d) and dorsal horn of the spinal cord (e, f). (c and f) are enlargements of the boxed areas in (b and e), respectively. Bars = 100 μm (d); 500 μm (e); 200 μm (f). (g–j) GRP expression in theTG and cervical DRG of adult male macaque monkeys. GRP-ir neurons were mainly observed in the small-sized TG neurons (g, h) and DRG neurons (i, j). (h) and (j) are enlargements of the boxed areas in (g) and (i), respectively. Bars = 200 μm (i); 50 μm (j). (k–r) Histograms of size distribution of GRP-positive and GRP-negative neurons in the monkey TG (k–n) and DRG (o–r). GRP-ir neurons were predominantly the small- and medium-sized TG and DRG neurons. GRP-positive TG neurons were 12% in the 8.3 kg female (k), 31% in the 7.2 kg female (I), 23% in the 2.7 kg male (m), and 25% in the 2.9 kg male monkey (n). GRP-positive DRG neurons were 31% in the 8.3 kg female (o), 15% in the 7.2 kg female (p), 20% in the 2.7 kg male (q), and 33% in the 2.9 kg male monkey (r). The average of GRP-positive TG neurons was 23% and DRG neurons was 25% in the four monkeys

The rabbit polyclonal GRP antiserum was raised against a 10-amino acid peptide sequence called neuromedin C or GRP-10. This antiserum produced identical patterns of labeling in the distribution of GRP-positive fibers in the trigeminal and spinal somatosensory systems as that achieved by IHC using the same (Satoh et al., 2015a; Takanami et al., 2014) or other antisera (Sakamoto et al., 2008) in rats. Control procedures for the 3,3′-diaminobenzidine (DAB) method were performed using preabsorption of the working dilution (1:2,000) of the primary antiserum with a saturating concentration of human GRP-10 antigen peptide, Gly-Asn-His-Trp-Ala-Val-Gly-His-Leu-Met (50 μg/mL, produced by AnaSpec, San Jose, CA) overnight at 4°C before use (Figure 2). The GRP-positive fibers were detected according to the above protocol for peroxidase IHC.

The mouse monoclonal antibody to calcitonin gene-related peptide (CGRP) (ab81887; Abcam, Cambridge, MA) was raised against rat *α-*CGRP. Preincubation with the immunizing peptide (Tocris Bioscience, Bristol, UK) blocked the IHC staining at 10^−4^ M in the ratTG (Eftekhari et al., 2010; Takanami et al., 2014). This antibody produces an identical staining pattern for the CGRPergic primary afferents in rats (Kruger et al., 1988; Sugimoto et al., 1997).

A guinea pig polyclonal antiserum (ab10353; Abcam) to peptide consisting of amino acids 1–11 of rat substance P was used. Preabsorption of the diluted antiserum (1:10,000) with 100 μg/mL of the antigen peptide (ab38217; Abcam) abolished staining in the rat spinal dorsal horn and DRG (Takanami et al., 2014). This antiserum produced patterns of labeling that were identical in populations of small- to medium-sized DRG and TG cells, and also identical to the patterns of primary afferent terminals in the spinal dorsal horn of rats derived from DRG cells, as reported by IHC using this same antiserum (Dhaka et al., 2008; Jeffry et al., 2009).

The GRPR antibody (GTX100015; GeneTex, Irvine, CA) was raised against synthetic peptide with human GRPR and displayed an intense band of approximately 43 kDa on Western blot of mouse and rat tissues (manufacture’s datasheet) and Asian house musk shrew, suncus Sp5C, and spinal dorsal horn (Takanami et al., 2016).

Anti-fluorescein isothiocyanate (FITC) antibody conjugated to horseradish peroxidase (HRP) (NEF710, PerkinElmer, Waltham, MA), which is not endogenous to vertebrate tissues, recognizes FITC and 5,6-carboxyfluorescein-labeled protein. Signals of fluorescein-labeled riboprobes were eliminated by preincubation of this antibody in the ferret brain sections to confirm the antibody specificity (Rowell et al., 2010). Full details of all antibodies used are presented in Table 1.

### 2.7 | IHC and immunofluorescence with frozen sections

We performed an IHC analysis according to our established methods using cryosections (Takanami et al., 2014; Takanami et al., 2021). In brief, tissue sections were first rinsed five times with PBS to remove the cryoprotectant. Endogenous peroxidase activity was eliminated by incubation in 1% H_2_O_2_ in absolute methanol for 20 min, followed by three 5-min rinses with PBS. This H_2_O_2_ treatment process was omitted in the immunofluorescence method. After blocking nonspecific binding with 1% normal goat serum and 1% BSA in PBS containing 0.3% Triton X-100 for 30 min at room temperature, sections were incubated with primary rabbit antiserum, which recognizes GRP-10 (11081–05015; AssayPro, St. Charles, MO) (1:2,000 dilution) for 2 days at 4°C. The GRP antiserum used in this study has previously been shown to be specific for GRP in the spinal cord of rats, mice, and Asian house musk shrews (Ito et al., 2018; Oti et al., 2021; Satoh et al., 2015a, b; Takanami et al., 2014; Tamura et al., 2017). Immunoreactive (ir) products were detected with a streptavidin-biotin kit (Nichirei, Tokyo, Japan), followed by DAB development according to our previous method (Takanami et al., 2014). GRP-immunoreactivity was imaged using an FSX100 microscope (Olympus, Tokyo, Japan) and all-in-one fluorescence microscope (BZ-H3XD, Keyence, Osaka, Japan). In addition, to determine the projection site of GRP-containing fibers, double immunofluorescence staining for GRP (1:1,000 dilution) and CGRP (1:20,000) (ab81887; Abcam), and substance P (1:10,000) (ab10353; Abcam) was performed as described previously (Takanami et al., 2014). Specificity of these antibodies was already confirmed using rat tissues (Takanami et al., 2014). Double-fluorescence staining of GRP and biotin-conjugated isolectin B_4_ (IB_4_) (1:1,000; cat. no. 121414; Molecular Probes, Eugene, OR) was also performed. IB_4_ from the plant *Griffonia simplicifolia* binds to small DRG neurons and dorsal horn lamina II inner (lli) layer through *α*-d-galactose carbohydrate residues on their soma membranes (Fang et al., 2006; Fullmer et al., 2004; Silverman & Kruger, 1990). Alexa Fluor 546-linked anti-mouse IgG (Molecular Probes), Alexa Fluor 546-linked anti-guinea pig IgG, Alexa Fluor 488-linked anti-rabbit IgG, and streptavidin-conjugated Alexa Fluor 546 were used at a 1:1,000 dilution for detection. Immunostained sections were imaged with a confocal laser scanning microscope (FluoView FV1000, Olympus). IHC studies were repeated independently at least three times using different animals and they produced similar results. The numbers and perikaryon sizes of GRP-positive and GRP-negative neurons with distinct nuclei were determined for both the TG and DRG. The soma size of TG neurons was calculated as the area of an ellipse (*π* × half the major axis length of the soma × half the minor axis length of the soma). The cross-sectional areas of TG neurons and cervical DRG neurons were measured in five sections from TG and DRG of each monkey. Image J was used to measure major axis length and the minor axis length of the soma. The numbers and sizes of cell bodies of both GRP+ and GRP− TGs and DRGs from each monkey are provided in Table 2. Histograms of cell body size of GRP-positive and GRP-negative neurons were made in 400 μm^2^ segments (Figure 2k–r).

### 2.8 | IHC with human paraffin sections

Paraffin-embedded tissue slices (5 μm in thickness) of human spinal cord (20-year-old male) were purchased from BioChain Institute (Catalog No.: T2234234; Lot No.: B101132) (Hayward, CA), as previously described (Takebe et al., 2013; Yabuki et al., 2007). After deparaffinization, sections were immersed in the Target Retrieval Solution (Dako S1700, Agilent, Santa Clara, CA) for 20 min at 98°C for antigen retrieval. After cooling at room temperature for approximately 30 min, the sections were washed in water. Then, endogenous peroxidase activity was eliminated from the sections by incubation in a 1% H_2_O_2_ absolute methanol solution for 20 min. After blocking nonspecific binding with 1% normal goat serum and 1% BSAin PBS containing 0.3% Triton X-100 for 30 min, sections were incubated in Can Get Signal A (Toyobo, Tokyo, Japan) with a 1:1,000 dilution of primary rabbit antiserum against NMC (AssayPro) for 24 h at 4°C. Ir products were detected with a streptavidin-biotin kit (Nichirei), followed by DAB development with 0.02% NiCl_2_. GRP-ir in the spinal cord were imaged using an Olympus FSX100 microscope. Control procedures for the DAB method were performed using preabsorption of the working dilution (1:1,000) of the primary antiserum with saturating concentration of human GRP_18–27_ (or GRP-10; corresponding to the human GRP-deduced amino acid residues) antigen peptide, Gly-Asn-His-Trp-Ala-Val-Gly-His-Leu-Met (50 μg/mL, produced in AnaSpec) overnight at 4°C before use.

### 2.9 | Tissue preparation for Western blot and Western ligand blot

Two females macaque monkeys were sacrificed by exsanguination under deep pentobarbital anesthesia. Brainstem and cervical spinal cords were quickly removed and placed on dry ice and were carefully dissected. Samples were snap-frozen immediately in liquid nitrogen and used for Western analyses.

### 2.10 | SDS-PAGE

Lysate samples for GRPR measurement (50 μg excised from the caudal part of the spinal trigeminal nucleus and cervical spinal dorsal horn protein using 18 G needle) were boiled in 10 μL sample buffer, containing 62.5 mM trishydroxymethyl-aminomethane-HCI (Tris-HCI; pH. 6.8), 2% SDS, 25% glycerol, 10% 2-mercaptoethanol, and a small amount of bromophenol blue. Samples were then separated by 4–20% gradient SDS-PAGE and electroblotted onto polyvinylidene difluoride (PVDF) membranes (Bio-Rad Laboratories, Hercules, CA) using a semidry blotting apparatus (Bio-Rad Laboratories).

### 2.11 | Western blot

Western blotting was conducted according to our previously described methods (Takanami et al., 2016). Membranes were blocked with the PVDF blocking reagent from the Can Get Signal kit (Toyobo) for 30 min at room temperature and then incubated overnight at 4°C in Can Get Signal Solution 1 (Toyobo) containing a 1:1,000 dilution of rabbit polyclonal antibody against human GRPR (GTX100015, GeneTex). Blotted membranes were washed three times with 0.05% Tween 20 in Tris-HCI buffered saline (TBST) and incubated with HRP-conjugated goat polyclonal antibody against rabbit IgG (Bio-Rad Laboratories) at a 1:10,000 dilution in Can Get Signal Solution 2 (Toyobo) for 1 h at room temperature. After washing five times with TBST, blots were visualized by the Immun-Star WesternC Chemiluminescence Kit (Bio-Rad Laboratories). Images of blots were detected by ChemiDoc XRS+ System with Image Lab Software (Bio-Rad Laboratories) and adjusted slightly for brightness and contrast to provide a uniform background.

### 2.12 | RNAscope in situ hybridization

RNA in situ hybridization was performed using RNAscope 2.5 HD Singleplex Reagent Kit (Advanced Cell Diagnostics, Hayward, CA) according to the manufacturer’s instructions for fresh frozen tissues. Chromogenic detection was performed using DAB followed by counterstaining with hematoxylin (131-09665, Fujifilm Wako Pure Chemical Corporation, Osaka, Japan). RNAscope probes for human *GRPR* (Hs-Grpr: 460411) were used. Stained sections were analyzed using a Nikon microscope.

### 2.13 | Western ligand blot

Western ligand blotting was conducted according to our previously described methods (Hasegawa et al., 2017; Hasegawa et al., 2020). Membranes were blocked with the PVDF blocking reagent (Toyobo) for 30 min at room temperature. After washing three times with TBST, the blot was probed with FITC-GRP-10 (Gly-Asn-His-Trp-Ala-Val-Gly-His-Leu-Met) at 0.34 μM for 30 min at room temperature. Blotted membranes were washed three times with TBST and incubated with HRP-conjugated goat polyclonal antibody against FITC (NEF710, PerkinElmer) at a 1:2,000 dilution in TBST for 1 h at room temperature. Detection of the signals was performed in a similar method as Western blot.

### 2.14 | Ligand derivative staining with rhodamine-GRP-10

Fresh frozen sections were blocked with Block One (Nacalai Tesque, Kyoto, Japan) for 30 min, and then incubated with rhodamine-GRP-10 (500 nM) in 20% Blocking One/TBST at room temperature for 30 min. After washing three times with TBST, DAPI (Sigma-Aldrich, St. Louis, MO, diluted 1:1,000) in PBS was incubated for 15 min. The sections were mounted with Fluoromount/Plus (Diagnostic BioSystems, Pleasanton, CA). Fluorescent and phase contrast images were observed with a microscope BX50 (Olympus).

### 2.15 | Statement of ethics

We certify that all applicable institutional and governmental regulations concerning the ethical use of animals were followed during the course of this research.

## 3 | RESULTS

### 3.1 | Sequence of *GRP* and *GRPR* in Japanese macaque monkeys

To verify the sequences of *GRP* and *GRPR* genes in Japanese macaque monkeys, we partially cloned cDNA encoding *GRP* from cervical DRG and *GRPR* from the dorsal horn of the spinal cord. Cloning data for macaque GRP were registered in the GenBank (GRP: LC619756). Using our cloning data for *GRP*, the amino acid sequence of *M*. *fuscata* GRP was highly conserved in mammals, especially in primates (Figure 1). The C-terminal amino acid sequence of GRP called the GRP-10 region, showed high similarity among mammals (Figure 1). We partially cloned the *GRPR* sequence of M. *fuscata* from two regions (GRPR: LC619165 and LC619166). Comparing the nucleotide sequences of cloned *M*. *fuscata* GRPR and M. *mulatto GRPR* obtained from the GenBank (NM_001278448.2), 99.6% homology in the 33-593 bp and 1686–1938 bp regions of *M*. *mulatto* was observed. These results showed GRP mRNA expression in the DRG and *GRPR* mRNA expression in the spinal dorsal horn in *M*. *fuscata*.

### 3.2 | Antibody specificity of GRP in the macaque somatosensory system

GRP was present in the somata of DRG neurons (Figure 2a) and present in the fibers and terminals within the spinal cord (Figure 2b, c). The distribution pattern of GRP-positive neurons was similar to that seen in mouse, suncus, and rat tissues (Fleming et al., 2012; Sun & Chen, 2007; Takanami et al., 2016, 2014). The specificity of the GRP antiserum was confirmed by control absorption experiments in which the primary rabbit antiserum against GRP-10 was preabsorbed with an excess of GRP-10 antigen peptide. This preabsorbed antiserum resulted in an absence of staining of GRP-ir neurons in DRG (Figure 2d) and spinal dorsal horn (Figure 2e, f).

### 3.3 | GRP-immunoreactivity in the macaque trigeminal and spinal somatosensory systems of both sexes

IHC analysis was performed to determine whether GRP, which has been reported to be expressed in rodents and Eulipotyphla, is localized in the trigeminal and spinal somatosensory system in primates. GRP-ir neurons were observed in relatively small-sized TG (Figure 2g, h) and DRG neurons (Figure 2i, j) of macaques. GRP-ir fibers with clear varicose structures were also observed in the TG (Figure 2h: arrowhead) and DRG (Figure 2j: arrowhead). No obvious sex difference was found in the expression patterns of GRP. The sensory modality transmitted by primary afferents is related to soma size distribution and associated axonal caliber and conduction velocity. The cell body areas of both female and male TGs and DRGs showed a bell-shaped distribution up to about 8,000 μm^2^ with a peak at 800–1,200 μm^2^ (Figure 2k–r). In summary, GRP-ir neurons were expressed in mainly small- and medium-sized TG (Figure 2k–n) and DRG neurons (Figure 2o–r). Next, the proportion and size of GRP-ir neurons in the TG and cervical DRG were analyzed. GRP-expressing TG neurons accounted for 12% (120/1,002 neurons: Figure 2k) in a female monkey (8.3 kg), 31% (339/1,110 neurons: Figure 2l) in a female monkey (7.2 kg), 23% (380/1,642 neurons: Figure 2m) in a male monkey (2.7 kg), and 25% (332/1,312 neurons: Figure 2n) in a male monkey (2.9 kg). GRP-expressing DRG neurons accounted for 31% (259/825 neurons: Figure 2o) in a female monkey (8.3 kg), 15% (95/635 neurons: Figure 2p) in a female monkey (7.2 kg), 20% (176/868 neurons: Figure 2q) in a male monkey (2.7 kg), and 33% (301/909 neurons: Figure 2r) in a male monkey (2.9 kg). The average percentage of GRP-positive neurons in the four monkeys was 23% in TG and 25% in DRG.

**FIGURE 3:**
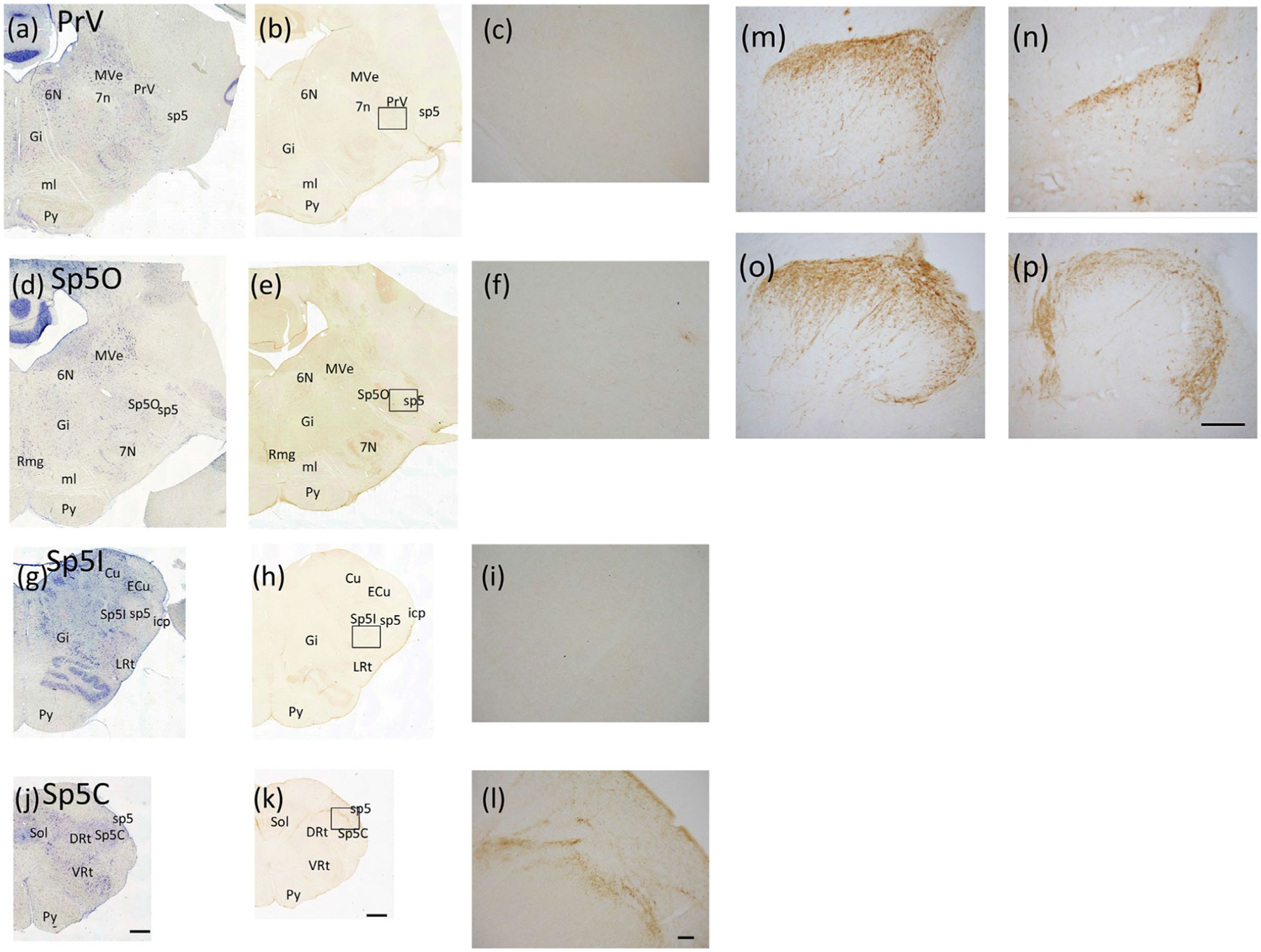
Distribution of GRP-ir fibers in the trigeminal nuclei and spinal dorsal horn of the adult macaque monkey, (a–l) Distribution of GRP-ir fibers in the trigeminal nuclei, (a, d, g, and j) show toluidine blue stained sections to visualize the localization of cell bodies. GRP-ir fibers were rare in the principal sensory trigeminal nucleus (PrV) (a–c),oral part of the spinal trigeminal nucleus (Sp50) (d–f), and interpolar part of the spinal trigeminal nucleus (Sp5l) (g–i). GRP-ir was dense in the caudal part of the spinal trigeminal nucleus (Sp5C) (j–l). (c, f, i) and (I) are enlargements of the boxed areas in (b, e, h) and (k), respectively. Bars = 1 mm (j, k); 100 μm (l).6N,abducens nucleus; 7N, facial nucleus; 7n, facial nerve; Cu, cuneate nucleus; DRt, dorsal reticular nucleus; ECu, external cuneate nucleus; Gi, gigantocellular reticular nucleus; icp, inferior cerebellar peduncle; LRt, lateral reticular nucleus; ml, medial lemniscus; MVe, medial vestibular nucleus; PrV, principal part of the trigeminal nucleus; py, pyramidal tract; RMg, raphe magnus nucleus; sp5, spinal trigeminal tract; Sp5C, caudal part of the trigeminal nucleus; Sp5l, interpolar part of the trigeminal nucleus; Sp50, oral part of the trigeminal nucleus; Sol, solitary nucleus; VRt, ventral reticular nucleus, (m–p) Distribution of GRP-ir fibers in the spinal dorsal horn of adult male macaque monkeys. GRP-ir fibers were observed in the cervical (m), thoracic (n), lumbar (o), and sacral (p) levels in the superficial laminae of the spinal dorsal horn. Bar= 200 μm (p)

Cranial TG neurons terminate in the trigeminal sensory nuclei of the medulla. The trigeminal sensory nuclei are structurally and functionally divided into the principal sensory trigeminal nucleus (PrV) and three subnuclei of the spinal trigeminal nucleus: oral part (Sp50); interpolar part (Sp5l); and caudal part (Sp5C) (Olszewski, 1950). Therefore, we next examined the localization of GRP-immunoreactivity in the macaque trigeminal nuclei. GRP-ir fibers were rarely observed in the PrV (Figure 3a–c), Sp50 (Figure 3d–f), and Sp5l (Figure 3g–i). In contrast, GRP-ir fibers were densely distributed in the macaque dorsal horn of the Sp5C (Figure 3j–l). Spinal DRG neurons terminate in all segmental levels of the spinal dorsal horn. GRP-ir fibers were densely distributed in the superficial layers of the spinal dorsal horn in the cervical (Figure 3m), thoracic (Figure 3n), lumbar (Figure 3o), and sacral level (Figure 3p) of males and females. No obvious sex difference in GRP staining patterns was observed in the trigeminal sensory nuclei and spinal dorsal horn.

**FIGURE 4:**
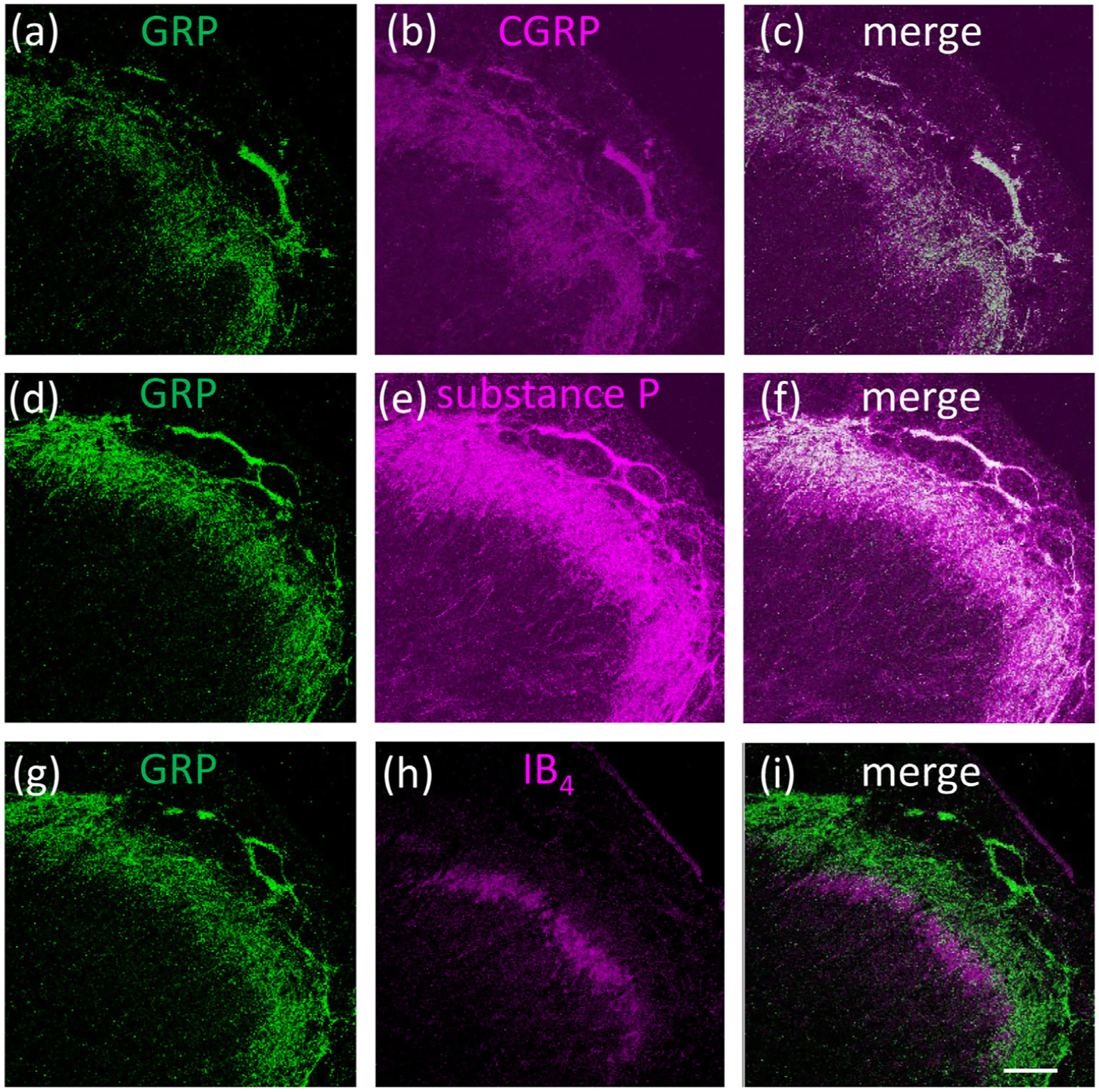

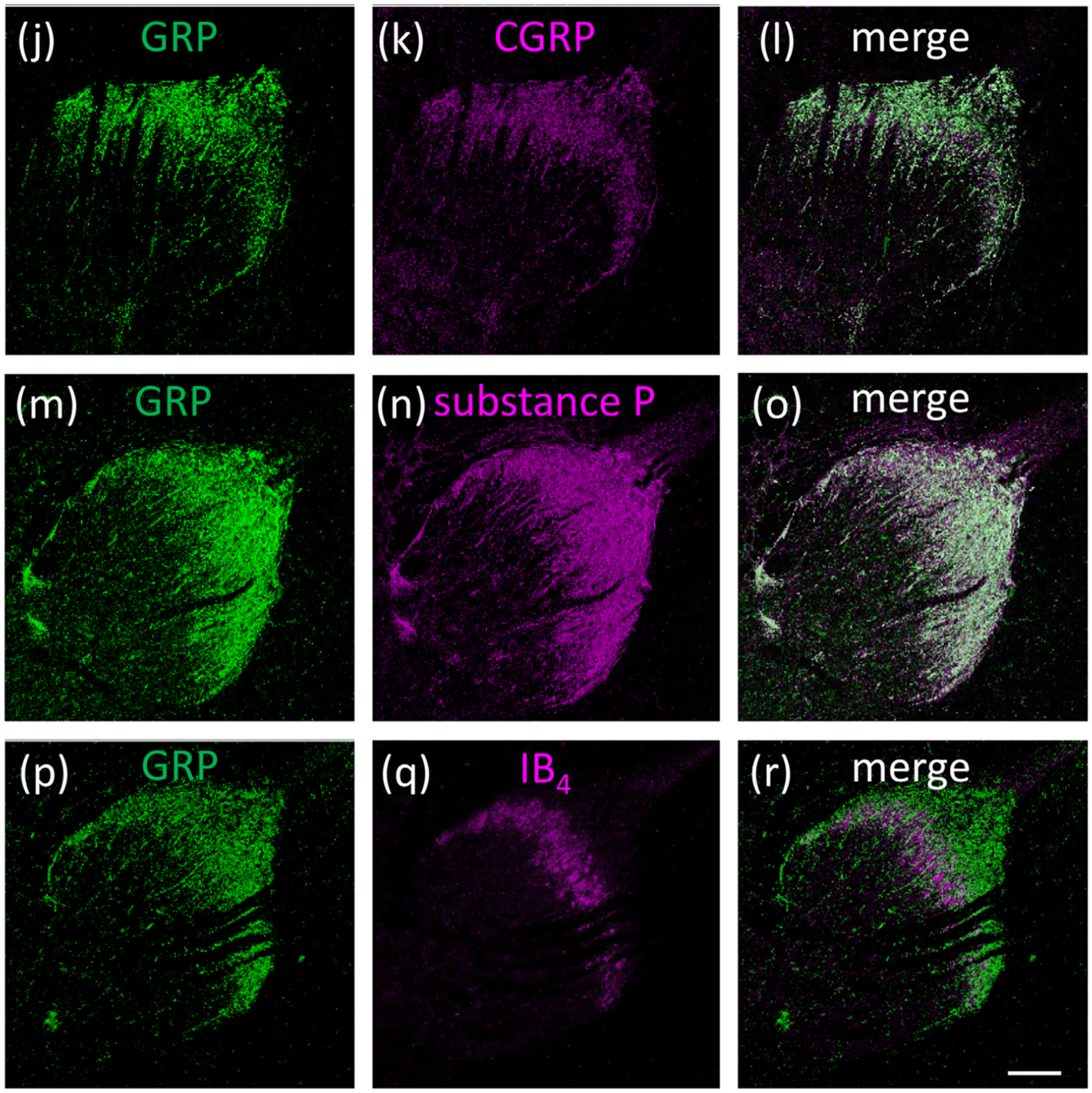
Double fluorescence of the GRP (green) and superficial layers markers (magenta) of Sp5C and cervical spinal dorsal horn in adult macaque monkeys. Many GRP-ir fibers were colocalized with the marker for peptidergic neurons CGRP (a–c) and substance P (d–f) in the Sp5C. GRP-ir fibers were not colocalized with the marker for nonpeptidergic neurons IB_4_ in the Sp5C (g–i). Most GRP-ir fibers were colocalized with CGRP(j–l) and substance P (m–o) in the superficial layers of the cervical spinal cord. GRP-ir fibers were not colocalized with IB_4_ (p–r) in the cervical spinal dorsal horn. Bars = 200 μm (i, r)

To investigate which laminae GRP-positive fibers project to in the Sp5C and spinal cord, double staining was performed for GRP and the major peptide markers of the primary afferents, CGRP and substance P, as well as the nonpeptide marker, IB_4_. GRP-ir fibers partially coexpressed CGRP in the Sp5C (Figure 4a–c) and cervical spinal dorsal horn (Figure 4j–l). GRP-ir fibers mostly coexpressed substance P in the Sp5C (Figure 4d–f) and cervical spinal dorsal horn (Figure 4m–o). In contrast, there was very little coexpression of GRP with IB_4_, markers of lamina Mi in the Sp5C (Figure 4g–i) and cervical spinal dorsal horn (Figure 4p–r).

### 3.4 | GRPR expression in the macaque trigeminal and spinal somatosensory systems

**FIGURE 5:**
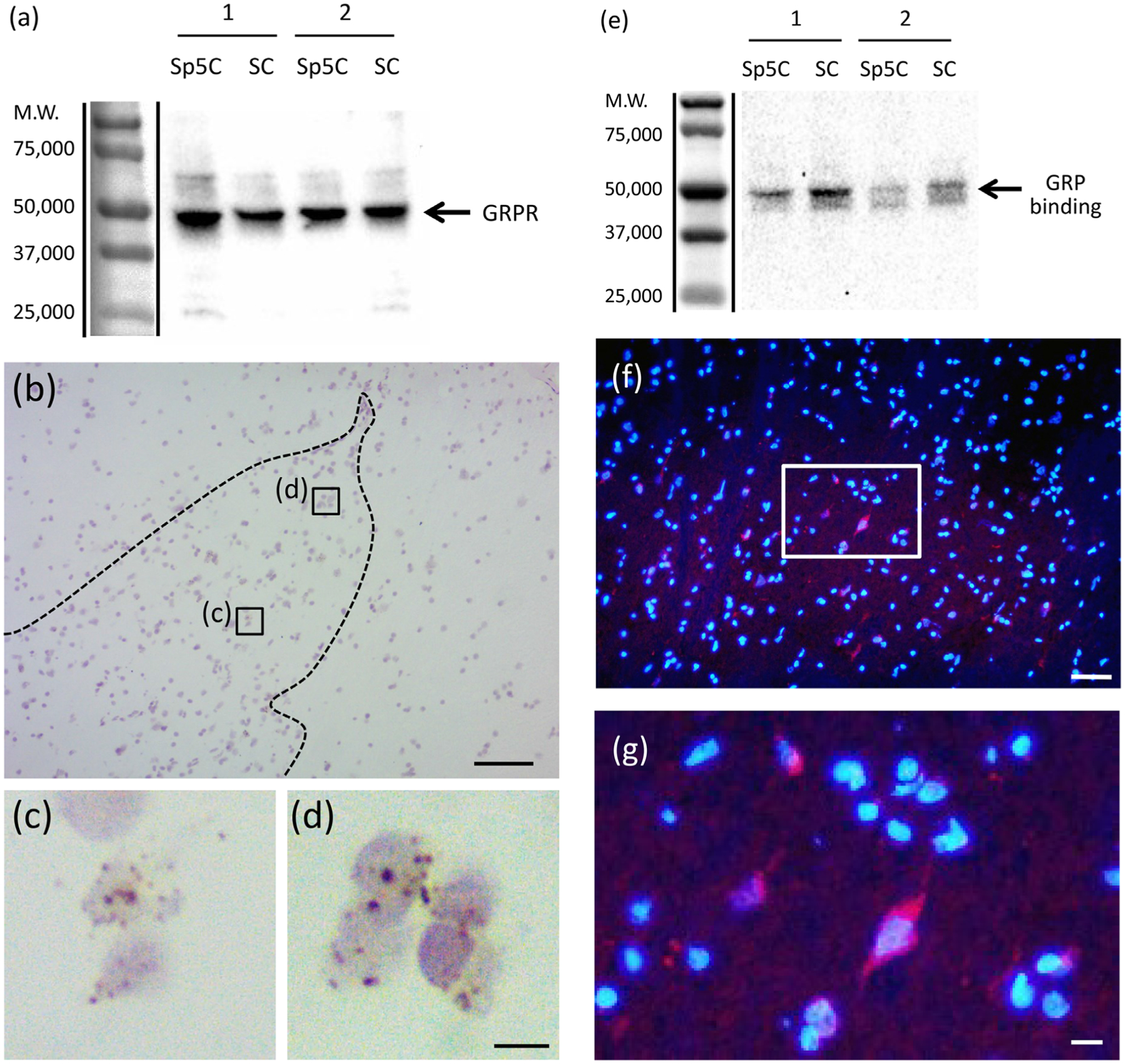
Expression of GRPR mRNA and GRPR protein and GRP binding sites in the sensory system of the adult macaque monkeys, (a) Expression of GRPR protein in the trigeminal and spinal cord. The number on the left indicates the molecular weight. The GRPR antiserum recognized a band at the expected molecular weight of GRPR (~43 kDa) on western blots of Sp5C and the dorsal horn of the cervical spinal cord (SC) of the macaque monkeys. Numbers 1,2 indicate different individuals. M.W. Molecular weight, (b–d) Localization of *GRPR* mRNA in the cervical spinal dorsal horn of the adult macaque monkey. *GRPR* mRNA was expressed in neurons within some superficial layers of the spinal dorsal horn (b–d). The reddish-brown dot structures were *GRPR* mRNA signals (c, d) and the nuclei were visualized in a light purple color by counterstaining with hematoxylin, respectively, (c and d) are enlargements of the boxed areas in (b), respectively. Bars = 100 μm (b); 10 μm (d). (e) Expression of the GRP-ligand binding sites in the trigeminal and spinal cord of the adult macaque monkeys. The number on the left indicates the molecular weight. The FITC-GRP-10 binding was recognized as an intense band at the expected molecular weight of GRPR (~43 kDa) on Western ligand blot of Sp5C and the dorsal horn of the cervical spinal cord (SC). Numbers 1,2 indicate different individuals. M.W. Molecular weight, (f, g) Ligand derivative staining with rhodamine-GRP-10 in the spinal cord of adult female macaque monkeys. Double staining of rhodamine-GRP-10 (red) and DAPI (blue). Rhodamine-GRP-10 signals were observed in a few neurons of the superficial layers of the cervical spinal dorsal horn, (g) is enlargements of the boxed area in (f). Bars = 50 μm (f); 10 μm (g)

Next, we analyzed the expression and GRPR localization in the macaque somatosensory system. Western blot analysis using anti-GRPR antiserum showed an intense band at approximately the expected molecular weight of GRPR (~43 kDa) in lysates prepared from the macaque dorsal horn of the Sp5C and cervical spinal dorsal horn (Figure. 5a). In situ hybridization revealed that GRPR mRNA was expressed in some cervical spinal dorsal horn neurons in the superficial layers in which the GRP-ir fiber projections were observed (Figure 5b–d).

### 3.5 | GRP-binding in the macaque trigeminal and spinal somatosensory systems

We examined whether GRP can bind to neurons in the macaque trigeminal and spinal somatosensory systems in which *GRPR* mRNA and GRPR protein expressions were observed. Western ligand blot revealed that fluorescent signals derived from FITC-GRP-10 were detected in the macaque Sp5C and cervical spinal dorsal horn lysates (Figure 5e). Fluorescent staining with rhodamine-GRP-10 also revealed that red fluorescent signals were observed in many neurons in the superficial layers of the macaque cervical spinal dorsal horn (Figure 5f,g).

### 3.6 | GRP expression in the human spinal cord

**FIGURE 6:**
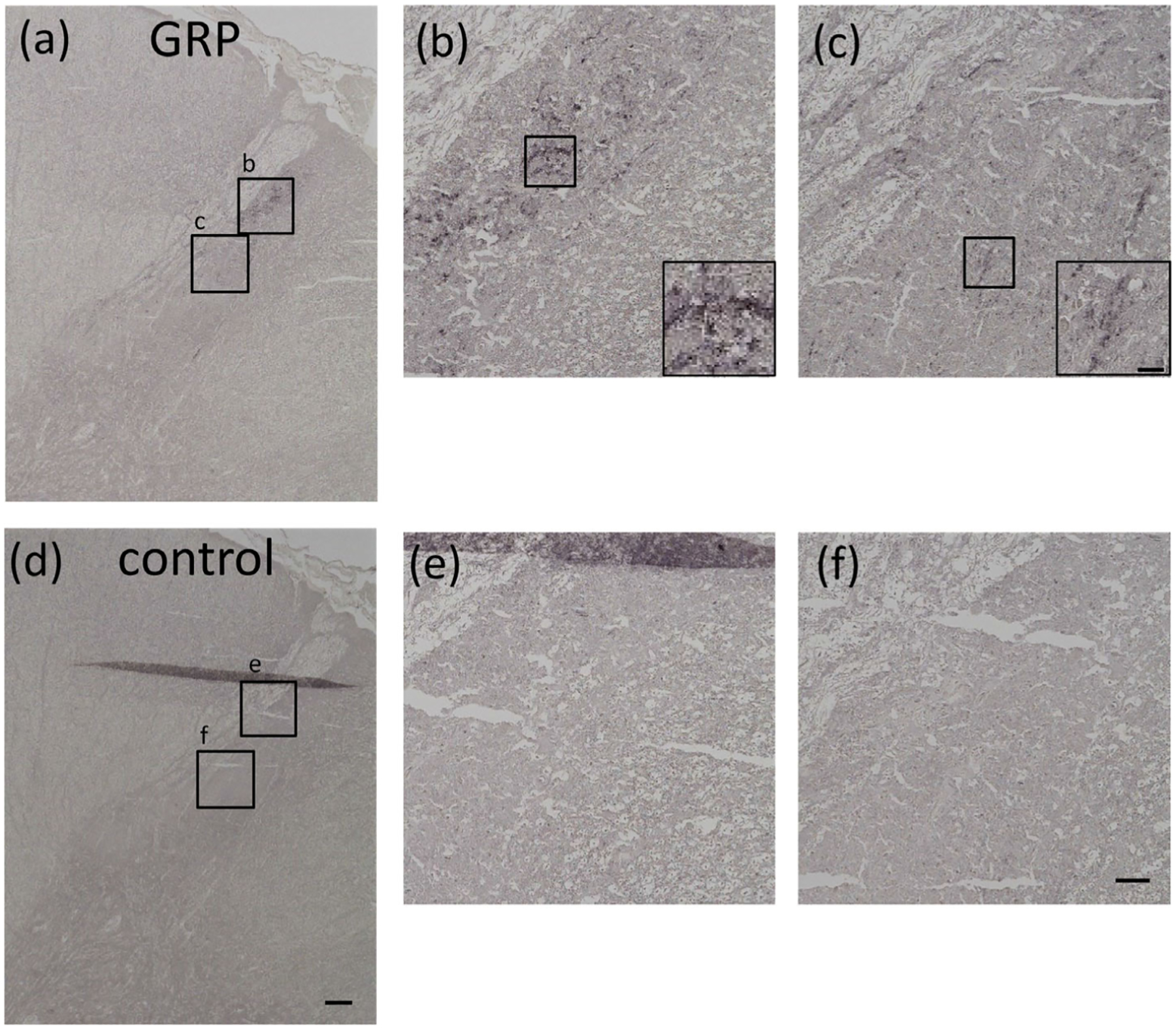
Immunohistochemical staining using GRP antiserum in the spinal cord in adult human male. GRP-ir was observed in dense fiber projections to the superficial layers of the transition area between thoracic and lumbar spinal cord (a–c). Controls in which anti-GRP antiserum was preabsorbed with an excess of antigen peptide (50 μg/mL) showed an absence of GRP expression in the spinal dorsal horn (d–f). (b, c, e, and f) are enlargements of the boxed areas in (a) and (d), respectively. Bars = 200 μm (d); 50 μm (f); 10 μm (inserted boxed area of c)

Finally, we analyzed the expression of GRP in the human spinal cord. GRP-ir fibers were present in the superficial layers of the transition area of the thoracic and lumbar spinal cord in the adult male (Figure 6a–c). The distribution pattern of GRP-ir fibers was similar to that seen in macaque monkeys. The specificity of the GRP antiserum reactivity was confirmed by control absorption experiments in which the primary rabbit antiserum against GRP-10 was preabsorbed with an excess of GRP-10 antigen peptide. This preabsorbed antiserum resulted in an absence of staining of GRP-ir neurons in the spinal dorsal horn (Figure 6d–f).

## 4 | DISCUSSION

This study shows for the first time that GRP is expressed in both TG and DRG neurons and GRPR is expressed in both the dorsal horn of the spinal trigeminal nucleus and spinal cord of Japanese macaque monkeys, similar to rodents and Eulipotyphla (Takanami et al., 2016, 2014). Furthermore, GRP-positive fibers in the spinal dorsal horn were also found in human tissue. These results suggest that in addition to spinal itch transmission from the lower body, the GRP-GRPR system may also transmit itch information from orofacial regions to the brain via the spinal trigeminal nucleus in primates.

We first found *GRP* mRNA was expressed in the TG and DRG of adult macaque monkeys, as previously described in rodents (Barry et al., 2020; Liu et al., 2014; Zhao et al., 2014). The amino acid sequence of GRP is well conserved among human and nonhuman primates reported so far. In particular, the GRP amino acid sequence shows a high homology among old world monkeys. The conservation of the GRP-10 amino acid sequence among mammals may indicate a link between molecular and functional evolution. For example, it is suggested that there is a need to conserve essential life maintenance functions, such as the transmission of itch in the somatosensory system, in the course of evolution. Recently, we reported that GRP and GRPR are expressed in the spinal cord in the amphibian *Xenopus* (Hirooka et al., 2021). Further discussion of the evolution of itch among vertebrates is needed.

From the present IHC analysis in Japanese macaques, GRP-positive neurons were present in the TG and projected to the superficial layers of the Sp5C. Many GRP-ir fibers coexpressed other peptidergic markers (e.g., CGRP and substance P) but not the nonpeptidergic marker, IB_4_, suggesting that GRP-expressing afferents from TG and DRG neurons terminate in the outer layers of the Sp5C and spinal dorsal horn (laminae l-ll). It has been reported that GRP is expressed in small- and medium-sized TG and DRG cells, as well as in dorsal horn neurons in rodents (Akiyama et al., 2014; Sun & Chen, 2007; Takanami et al., 2014). We presently show that the average percentage of GRP-positive neurons was 23% in TGs and 25% in DRGs and was similar in females and males. The sizes of the GRP-positive neurons are around 600 μm^2^, and thus smaller than GRP-negative neurons that are around 1,000 μm^2^ in monkey TGs and DRGs. Our data showed that GRP was particularly expressed in the small- and medium-sized primary afferents in monkeys similar to rodents (Akiyama et al., 2014; Sun & Chen, 2007; Takanami et al., 2014). We previously identified a sexually dimorphic GRP system in the sacral autonomic nucleus in the lumbosacral spinal cord of macaques that regulated penile function, but not in the lumbosacral spinal dorsal horn GRP system (Ito et al., 2018). In the present study, we found that GRP-ir fibers exist at all levels of the spinal cord and caudal part of medullary dorsal horn, and the expression pattern is similar between sexes. These results show that even in the same spinal cord region, GRP plays distinct roles in itch transmission as well as male sexual function with evidence for sexual dimorphism in the latter but not the former (Takanami & Sakamoto, 2014).

A previous study reported an increased number of GRPR-positive cells in the superficial layers of the spinal dorsal horn of macaque monkeys that exhibited greater itch severity (Nattkemper et al., 2013). We found that *GRPR* mRNA and GRPR protein were expressed in the Sp5C and dorsal horn of the spinal cord. Consistent with these results, the GRPR agonist (GRP-10) was shown to bind to neurons in the macaque Sp5C and spinal dorsal horn as shown by Western ligand blot and ligand derivative stain. It is reported that GRPR-expressing neurons in the spinal dorsal horn transmit the sensation of itch from the periphery to the brain in rodents (Kiguchi et al., 2020; Sun & Chen, 2007). In macaque monkeys, robust scratching behavior was dose-dependently elicited by intrathecal injection of GRP, in a manner that was inhibited by GRPR antagonists (Lee & Ko, 2015), similar to previous results in mice (Sukhtankar & Ko, 2013). Moreover, GRP induced itch behavior but did not have antihyperalgesiceffects in macaques (Lee & Ko, 2015). Considering the results of previous data and our data, GRP and GRPR expression in the trigeminal sensory system as presently reported suggests that the trigeminal GRP-GRPR system also appears to transmit itch information in primates.

It was difficult to find specific antibodies for GRPR for the present IHC analysis. The antibody that we selected was successfully used for Western blot, but however, not for IHC. For this reason, we used a ligand derivative stain as an alternative to IHC. The ligand derivative stain and Western ligand blot rely on the native binding of ligands to their specific receptors. In the Western ligand blot, the band that appeared was of a similar size as in the Western blot, consistent with the predicted molecular weight of GRPR. These results strongly argue for the specificity of the ligand binding to GRPR. In addition, the ligand labeled with rhodamine was used to detect GRPR-expressing cells in frozen sections. The Western ligand blot and ligand derivative stain proved to be useful when specific antibodies to receptors could not be found. It has been reported in humans that GRP-ir nerves are primarily located in the papillary dermis as free nerve endings terminating at the dermoepidermal junction of the scalp, head, neck, trunk and extremities (Timmes et al., 2013) similar to mice (Tominaga et al., 2009). Furthermore, GRP-positive fibers were observed in superficial layers of the spinal dorsal horn in the human spinal cord, similar to the distribution observed in macaque monkeys in this study. This suggests that the spinal GRP system is conserved in primates, although we did not confirm the GRP and GRPR expression in the trigeminal sensory system in humans. A recent study described the isolation of human *GRPR* in the spinal dorsal horn (Liu et al., 2019). The human μ-opioid receptor 1Y is required for cross-activation of human GRPR-mediated itch transmission (Liu et al., 2019) similar to the mouse p-opioid receptor 1D-GRPR cross-talk in the spinal cord (Liu et al., 2011). These and the present data imply the functional conservation of the GRP-GRPR system in mediating itch in mice and humans. The incidence of allergic pruritus such as pollen allergy, airborne particulate matter allergy, and atopic dermatitis appears to have significantly increased in recent years, negatively impacting the quality of life. Therefore, it is important to investigate the involvement of the spinal and trigeminal somatosensory GRP-GRPR system in the transmission of human itch to establish the neural mechanisms and novel treatments for chronic pruritus.

## Figures and Tables

**TABLE 1:** Primary antibodies used in this study

Antigen	Description	Host species and type	Working dilution	Catalog information	RRID
GRP	Mouse neuromedin C (or GRP-10; GSHWAVGHLM)	Rabbit polyclonal IgG	1:2,000 (IHC) 1:1,000 (IF)	AssayPro, 11081-05015	AB_2571636
CGRP	Synthetic rat *α*-CGRP, Clone 4901	Mouse monoclonal IgG	1:20,000 (IF)	Abcam, ab81887	AB_1658411
Substance P	Amino acid: 1–11 (RPKPQQFFGLM) of rat substance P	Guinea pig polyclonal IgG	1:10,000 (IF)	Abcam, ab10353	AB_297089
GRPR	Amino acids: 200–294 of human GRPR	Rabbit polyclonal IgG	1:1,000 (WB)	GeneTex, GTX100015	AB_1240922
FITC	FITC antibody conjugated to HRP	Sheep polyclonal IgG	1:2,000 (WLB)	PerkinElmer, NEF710	AB_2314403

Abbreviations. FITC, fluorescein isothiocyanate; HRP, horseradish peroxidase; IF, immunofluorescence; IHC, immunohistochemistry; WB, western blot; WLB, western ligand binding blot.

**TABLE 2:** Numbers and sizes (±SEM) of TG and DRG cells

Monkey	#TG cells (number)	GRP+TGcell body size (μm^2^)	GRP−TG cell body size (μm^2^)	#DRG cells (number)	GRP+ DRG cell body size (μm^2^)	GRP− DRG cell body size (μm^2^)
Female (8.3 kg)	1,002	930 ± 33	1,544 ± 23	825	899 ± 27	1,830 ± 58
Female (7.2 kg)	1,110	591 ± 16	1181 ± 21	635	675 ± 39	1,566 ± 47
Male (2.7 kg)	1,642	622 ± 13	1037± 22	868	567 ± 26	1,398 ± 43
Male (2.9 kg)	1,312	557 ± 14	1,126 ± 25	909	957 ± 27	2,001 ± 61

## Data Availability

The data that support the findings of this study are available from the corresponding author upon reasonable request.
